# The effect of varying monocalcium phosphate and polylysine levels on dental composite properties

**DOI:** 10.1016/j.jmbbm.2023.106039

**Published:** 2023-09

**Authors:** Nabih Alkhouri, Wendy Xia, Paul Ashley, Anne Young

**Affiliations:** a Department of Biomaterials and Tissue Engineering, UCL Eastman Dental Institute, London, NW3 2QG, UK; b Department of Paediatric Dentistry, UCL Eastman Dental Institute, London, WC1E 6DE, UK

**Keywords:** Dental composite, Polymerisation, Strength, Water sorption, Polylysine, Monocalcium phosphate

## Abstract

**Objectives:**

The aim was to quantify effects of polylysine (PLS, 2 or 5 wt%) and monocalcium phosphate (MCP, 4 or 8 wt%) on properties of dental composites.

**Methods:**

Light-activated, lower surface polymerisation kinetics versus sample depth (1–4 mm) of 4 formulations were quantified using ATR-FTIR. Water sorption and solubility (at 1 week) were assessed following ISO/4049. PLS release (over 1 month) and biaxial flexural strength (over 6 months) of fully-cured, water-immersed, 1 mm thick discs were determined**.** Surface mineral precipitation, following immersion in simulated body fluid (SBF), was assessed by SEM. Z250 was used as a conventional composite comparator.

**Results:**

With 40s light exposure, increasing depth (from 1 to 4 mm) led to enhanced delay before polymerisation (from 3 to 17s) and decreased final conversion (72-66%) irrespective of PLS and MCP level. Increasing PLS and MCP raised solubility (4–13 μg/mm^3^). Water sorption (between 32 and 55 μg/mm^3^) and final PLS release (8–13% of disc content) were raised primarily by increasing PLS. Higher PLS also reduced strength. Strength reached minimum values (69–94 MPa) at 3 months. Surface mineral deposition was enhanced by increased MCP. For Z250, polymerisation delays (3-6s) and final conversions (55-54%) at 1-4 mm depth, solubility (0 μg/mm^3^), water sorption (16 μg/mm^3^) and strength (180 MPa) were all significantly different.

**Conclusion:**

Delay time increased whilst final conversion decreased with thicker samples. Higher PLS enhances its percentage release, but lower level is required to keep water sorption, solubility and mechanical properties within ISO 4049 recommendations. Doubling MCP raises solubility and enhances minerals reprecipitation with minimal mechanical property compromise.

## Highlights

• Modified Renewal MI formulations have sufficient monomer conversion.
• The novel additive MCP can promote surface mineral precipitation.
• Higher PLS caused a significant decline in strength, but raised MCP did not.
• Higher MCP and intermediate PLS levels are optimal for a final formulation.

## 1 Introduction

Public Health England figures showed 12 and 23% of 3 and 5-year-old UK children respectively, have caries affecting 3–4 teeth (England, 2018). These teeth are mostly unrestored and adversely affect quality of life. Amalgam is now banned following the Minamata agreement, (FDI, 2014). The main alternative is the dental composite, but these require complex etch and bond procedures to ensure retention. These long steps are one of the reasons for the low proportion of restored children's teeth. Whilst with adult teeth, composite failure is usually a consequence of fracture due to inadequate strength, in children's teeth it is usually due to recurrent caries at the bonded composite restoration margin (Chisini et al., 2018) (Heintze et al., 2022). Continuing bacteria and enzyme catalysed decay may be enhanced by lack of composite antibacterial action (unlike amalgam) or ineffective sealing (Kim et al., 2017).

To address the above problems, new self-sealing composites have been developed and an optimised formulation, Renewal MI, recently manufactured (Alkhouri et al., 2022). Its main application is to restore children's teeth in an atraumatic manner. Laboratory studies have proven Renewal MI can, on its own, etch enamel and effectively seal drilled cavities (Alkhouri et al., 2022). Furthermore, it can stabilise demineralised dentine by penetrating several hundred microns into the open tubules. This enabled more effective inhibition of enzyme catalysed collagen degradation than observed with a conventional composite with a bonding agent or glass ionomer cements (Alkhouri et al., 2022). Additionally, Renewal MI promoted mineral precipitation from simulated body fluid at the demineralised dentine / restoration interface or within material cracks which may help to maintain cavity seal (Alkhouri et al., 2022). In a recent first-in-human clinical trial, these features enabled Renewal MI to effectively seal and be retained within highly carious minimally prepared cavities with no drilling or separate etchant or adhesive steps (Appendix H in reference (Alkhouri, 2020)).

Renewal MI is a light cured composite. It's high molecular weight urethane dimethacrylate (UDMA) / polyproplene dimethacrylate (PPGDMA) monomer phase enables rapid high polymerisation with low shrinkage (Walters et al., 2016). It also contains the adhesion promoting monomer, 4-methacryloyloxyethyl trimellitate anhydride (4META). Furthermore, low levels of two novel components, monocalcium phosphate (MCP) and polylysine (PLS) particles, have been added. Both have FDA Generally Recognised as Safe (GRAS) status and are used extensively in food. Upon MCP dissolution in water, it can disproportionate to form phosphoric acid and dicalcium phosphate (Alkhouri, 2020). Whilst hydrogen ions from the acid enable self-etch of enamel, the calcium and phosphate ions are required for tooth remineralisation. Reaction with and / or absorption of surface water, by the hydrophilic PLS and MCP particles, allows the hydrophobic resin to penetrate open dentine tubules. Furthermore, removal of water from the dentine halts its enzyme catalysed hydrolysis. Water sorption also induces composite swelling which may compensate polymerisation shrinkage (Alkhouri, 2020). Early PLS release from composites can kill planktonic Streptococcus Mutans and reduce biofilm development (Lygidakis et al., 2020; Yaghmoor et al., 2020).

A potential problem with higher levels of MCP and / or PLS, however, could be a reduction in polymerisation rates, increased water sorption and solubility, and reduced mechanical properties (Aljabo et al., 2015; Delgado et al., 2021).

The following study provides the key evidence employed to determine what levels of MCP and PLS could be included in Renewal MI without water sorption and solubility causing excessive decline in strength.

The null hypotheses are that changing the levels of MCP and / or PLS has no significant effect on:

1) final conversion upon varying depth,
2) water sorption and solubility at 7 days,
3) PLS release in the first month,
4) flexural strength over 6 months, or
5) minerals precipitation.

Additional null hypotheses tested are that these properties are not significantly different from those of a conventional dental composite, Z250.

## 2 Materials and methods

### 2.1 Materials and sample preparation

Composites produced in this study had identical composition to Renewal MI (Davis, Schotlander and Davis, Letchworth, UK) except for its percentages of MCP and PLS. Composite fillers consisted of silane-treated, radiopaque barium aluminosilicate glass of 7 μm and 0.7 μm (GM27884, Schott, Germany, lots 020684/680326, 02110/688344) with fumed silica (Aerosil OX 50, Evonik, Germany, lot 153022145) in the weight ratio 6:3:1. These were mixed with PLS of 20-50 μm (5 or 2 wt%) (Handary, Belgium, Epolyly lot 020120160203) and MCP 53 μm (8 or 4 wt%) (Himed, USA, lot 369925) particles. The clear yellow liquid phase consisted of UDMA (72 wt%) (DMG, Germany, lot 100112/97406), PPGDMA (24 wt%) (Polysciences, USA, lot 626208), 4META (3 wt%) (Polysciences, USA, lot 697058) base, diluent and acidic monomers with the initiator, camphorquinone, CQ (1 wt%) (DMG, Germany, lot 100134/90339) added.

Powders (75 wt%) were mixed with liquid (25 wt%) for 40 s in a speed mixer (Synergy speed mixer, UK) at 3500 rpm. Formulations were given the code Fxy where x and y were the PLS and MCP weight percentages in the filler, respectively (Table 1).

**Table 1:** Experimental formulations codes and composition.

Code^a^	Liquid (wt%)				Powder (wt%)		
	UDMA	PPGDMA	4META	CQ	Glass	PLS	MCP
F58	72	24	3	1	87	5	8
F54	72	24	3	1	91	5	4
F28	72	24	3	1	90	2	8
F24	72	24	3	1	94	2	4

Abbreviations: UDMA (Urethane dimethacrylate), PPGDMA (polypropylene glycol dimethacrylate), 4META (4-Methacryoyloxy trimellitate anhydride), CQ (Camphorquinone), PLS (Polylysine), MCP (monocalcium phosphate monohydrate).

a Formulations were given the code Fxy where x and y were the PLS and MCP weight percentages in the filler, respectively.

The commercial comparator was Filtek Z250 (Shade B3, 3M ESPE, USA, lot N851179).

To prepare discs, pastes were placed inside metal circlips (10 or 16 mm inner diameter and 1 mm thickness) with acetate sheet top and bottom. Each disc was light cured for 40s on each side using an LED curing gun (wavelength range 450-470 nm power output 1000 mW/cm^2^, Demi Plus, Kerr, USA). The light tip was placed in direct contact with the acetate sheet. Slow rotation (∼10 revolutions / minute) of the gun was employed to ensure the whole surface of samples was exposed to light. Light guide tip was 6 versus 13 mm diameter for small versus bigger discs. This enabled the specimen centre and edge to be simultaneously exposed during light tip rotation for both specimen sizes. Set samples were stored dry for 24 h before use.

### 2.2 Light curing kinetics

To assess polymerisation kinetics of composite pastes on the lower surface of samples away from the light source, an Attenuated Total Reflectance attachment (ATR, Specac Ltd., UK) in an FTIR spectrometer (Perkin Elmer, UK) was employed (Aljabo et al., 2015; Delgado et al., 2021; Young et al., 2004; Delgado and Young, 2021). Composite paste was packed into 1 or 4 stacked circlips each of 1 mm thickness and 10 mm diameter and centred on the ATR diamond (n=3). Sample top surfaces were covered with acetate sheet and spectra (700–4000 cm^−1^ with resolution of 4 cm^−1^) of the lower sample surface generated every 2.5s for 15 min at 24°C. The tip of the LED curing gun (6 mm guide) was placed at the centre of the sample and FTIR diamond (in direct contact with the top acetate sheet). At 30s it was turned on for 40s to initiate paste cure.

Conversion versus time was calculated using equation (England, 2018)

(1) $$C\left(\%\right)=100\left(h_{0}-h_{t}\right)/h_{0}$$

h_0_ and h_t_ represent the methacrylate C–O peak height at 1320 cm^−1^, above background at 1335 cm^−1^, initially and at time t (Aljabo et al., 2015; Delgado et al., 2021; Young et al., 2004; Delgado and Young, 2021). Final conversion was determined through linear extrapolation of conversion versus inverse time to zero. This method has previously been successfully used to assess maximum methacrylate conversion in a wide range of different formulations (Aljabo et al., 2015; Delgado et al., 2021; Young et al., 2004; Delgado and Young, 2021).

### 2.3 Water sorption and solubility

Water sorption and solubility (n=6) were determined using 16 mm diameter, 1 mm thick discs following ISO 4049-2009 (BSI, 2009). Briefly, discs were dried to constant initial mass (m_1_) in a desiccator at 37°C. Initial volume (V) was measured, then each specimen was immersed in 10 mL deionised water at 37°C for seven days and weighed again (m_2_). Samples were re-dried and reweighed to obtain final mass (m_3_).

The equations used to calculate water sorption and solubility (in μg/mm^3^) were:

(2) $$W_{sp}=\left(m_{2}-m_{3}\right)/V$$

(3) $$W_{sl}=\left(m_{1}-m_{3}\right)/V$$

### 2.4 Polylysine release

For PLS release quantification composite discs (10 mm diameter and 1 mm thick (n=3)) were immersed in 1 mL of deionised water at 37°C. At each time point (1 h, 3 h, 6 h, 1 day, 2 days, 3 days, 1 week, 2 weeks and 1 month), discs were moved into a fresh 1 mL of deionised water. High performance liquid chromatography (HPLC, Shimadzu) was used with a Luna® NH2 Column (250 x 4.6 mm, particle size of 5 μm, Phenomenax, Torrance, CA, USA) at 30°C. Injection volume was 200 μL, mobile phase was 50/50 acetonitrile/water with 0.1 vol% phosphoric acid and flow rate 1 mL/min. Retention time was 20 min and detection wavelength 210 nm. PLS solutions of 10–100ppm were used for calibration (Alkhouri, 2020).

Cumulative release of PLS was calculated using:

(4) $$R\left(\%\right)=100\left[\sum_{0}^{t}R_{t}\right]/m$$

where m is the weight of PLS powder in the sample, and $R_{t}$, amount leached into water at each time point t.

### 2.5 Biaxial flexural strength

Composite discs of 10 mm diameter and 1 mm thick (n=8 per formulation and time) were prepared and tested dry and after 1 day, 1 week, 1 month, 3 months and 6 months of immersion in 10m deionised water at 37°C. A Shimadzu Autograph, with a ball on ring jig, was used with crosshead speed of 1 mm/min.

Biaxial flexural strength (S) was calculated using (Higg et al., 2001):

(5) $$S=\frac{3\left(1+v\right)F}{4\pi h^{2}}\left\{1+2\;\ln\left(\frac{a}{b}\right)+\frac{\left(1-v\right)}{\left(1+v\right)}\left[1-\frac{b^{2}}{{2a}^{2}}\right]\frac{a^{2}}{r^{2}}\right\}$$

F is the force at break, h, sample thickness, v, Poison's ratio (0.3), a, radius of the support ring (4 mm), b, radius of the loading contact (assumed to be h/3 based on the Hertzian theory), r, radius of the sample (Higg et al., 2001).

### 2.6 Surface mineral precipitation

To assess mineral deposition, 10 mm diameter samples of 1 mm depth (n=1) were placed in 10 mL of simulated body fluid (SBF) (Implants, 2014) for 1 week. The surfaces were then examined using SEM (Phillip XL-30, Eindhoven, The Netherlands). Additionally, the fracture sites of 2 samples of F54 after 3 months of immersion in water were examined. These samples did not completely break upon mechanically testing. They were therefore examined by SEM, after further storage in SBF for 2 months, to assess if the sites could re-seal through mineral precipitation.

### 2.7 Statistical analysis

All properties and error bars reported are the mean with 95% confidence intervals. Factorial analysis with two variables (each at high and low levels) was used to calculate the effect of different variables on composite properties (Mehdawi et al., 2013). The following equation was used to calculate the percentage increase in a property caused by a variable:

(9) $$Increase\%=100(g_{h}{-g}_{l})/g_{l}$$

where $g_{h}$ and $g_{l}$ are the geometric mean property of samples sharing the same high or low level, respectively, of a variable.

SPSS Statistics version 24 for Windows (IBM, USA) was used for statistical analysis. To test significance (p<0.05) of variables and interactions on the properties, general linear models were used. When comparing the experimental composites to the commercial comparator Filtek Z250, Levene's test was used to assess homogeneity of variance. When variances were equal, data were analysed using one-way analysis of variance (ANOVA) followed by post-hoc Tukey's test for multiple comparisons when needed. Kruskal-Wallis test was used if variances were not equal followed by pairwise comparisons if needed (Yan et al., 2017).

## 3 Results

### 3.1 Light-curing kinetics

**Fig. 1:**
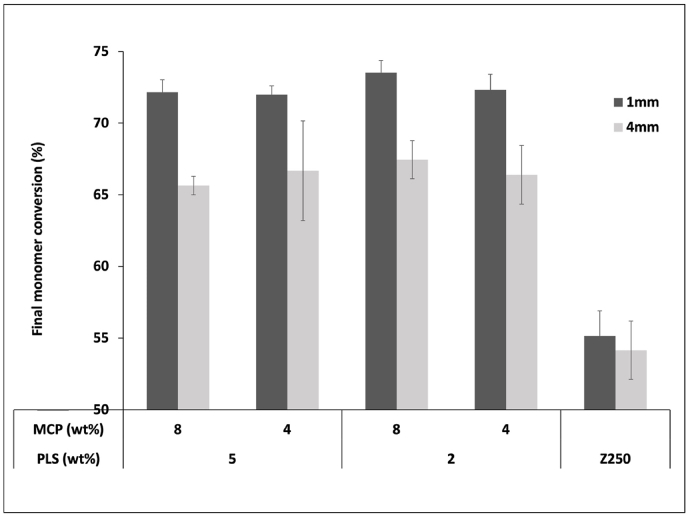
Maximum final monomer conversion percentage of all experimental formulations in comparison to Z250 (1 versus 4 mm thick samples). Error bars are 95% CI (n=3).

With 40s light exposure, there was a delay before any monomer conversion of 3±0 versus 17±1s, whilst extrapolated maximum final monomer conversion decreased slightly from 72±1 to 66±2% at 1 versus 4 mm depth, respectively (Fig. 1). These results were not significantly affected by PLS or MCP content. Z250, delay time increased from 3 to 6s whilst final conversion was 55 and 54% at 1 versus 4 mm depth. Final monomer conversion of Z250 was significantly lower than for the experimental formulations, regardless of the thickness (p<0.05).

### 3.2 Water sorption and solubility

**Fig. 2:**
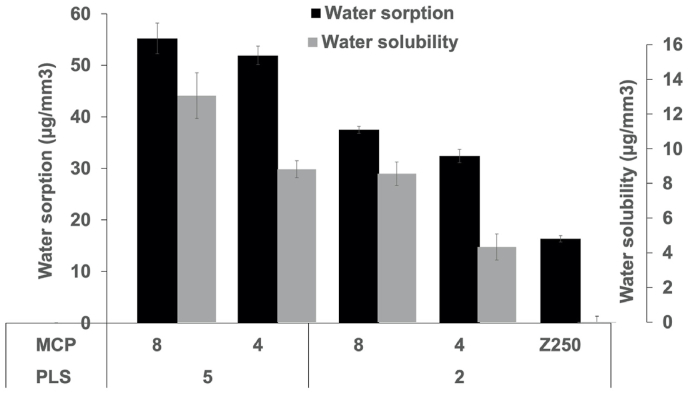
Water sorption and solubility of 16 mm diameter discs after 1 week water immersion. Error bars are 95% CI (n=6).

Water sorption at 1 week for experimental composites ranged from 32 to 56 μg/mm^3^ (Fig. 2). Factorial analysis showed water sorption was increased by 54% with higher PLS but by only 11% with higher MCP (p<0.05). Z250 showed significantly lower water sorption (16 μg/mm^3^) than all experimental formulations. F28, F24 and Z250 all had water sorption below the ISO 4049 maximum (40 μg/mm^3^).

For experimental materials, 1 week water solubility ranged from 4 to 13 μg/mm^3^ (Fig. 2). Factorial analysis showed this increased by 78% with higher PLS and by 73% with higher MCP (p<0.05). The water solubility for Z250 was negligible and significantly lower than all experimental composites (p<0.05). Solubility below the ISO limit (7 μg/mm^3^) was achieved only by F24 and Z250.

### 3.3 Polylysine release

**Fig. 3:**
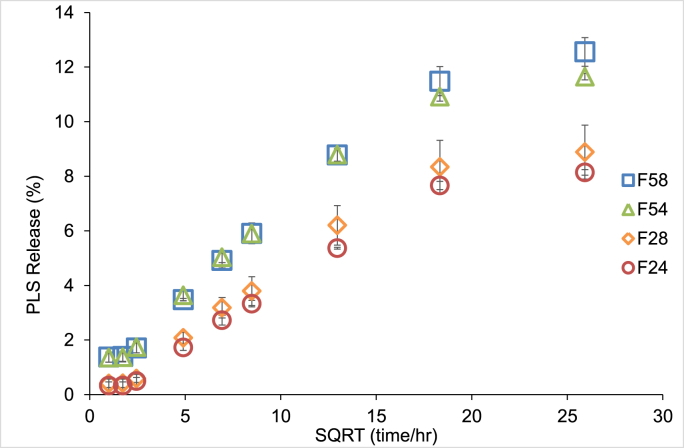
Cumulative Polylysine release percentage versus Square Root (SQRT) of time at 37°C for up to 1 month. Blue/green colours indicate high PLS, square/diamond indicate high MCP. Error bars are 95% CI (n=3).

Cumulative polylysine release was initially proportional to the square root (SQRT) of time reaching values of 6 and 9% of that in the materials by 1 week for low versus high PLS respectively. It levelled after 2 weeks (SQRT (time/hr)=18) (Fig. 3). The final PLS release after one month ranged between 8% and 13% of that in the samples. Factorial analysis showed that final PLS release was increased by 56% with higher PLS and 18% with higher MCP (p<0.05).

### 3.4 Biaxial flexural strength

**Fig. 4:**
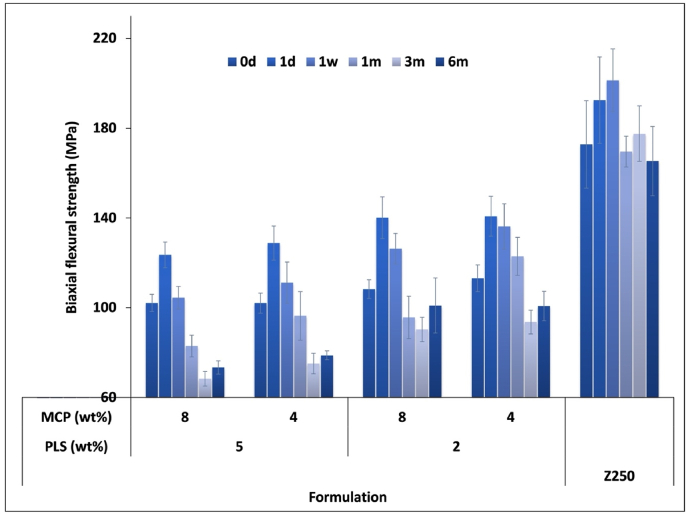
Biaxial flexural strength of the experimental formulations after immersion in water at 37°C up to 6 months. Error bars are 95% CI (n=8).

Flexural strength was a maximum at 1 day and between 124 and 141 MPa. It declined to constant, final values after 3 months that were between 73 and 101 MPa (Fig. 4). Over time, effects of PLS increased whilst that of MCP increased to a significant level at 1 month (p<0.05) then decreased. Strength at 6 months was 32% higher with reduced PLS (p<0.05) but not significantly affected by MCP level. Flexural strength of Z250 (180 MPa on average) was not greatly affected by time and significantly higher than that of all experimental formulations at any given time point (p<0.05).

### 3.5 Surface mineral precipitation

**Fig. 5:**
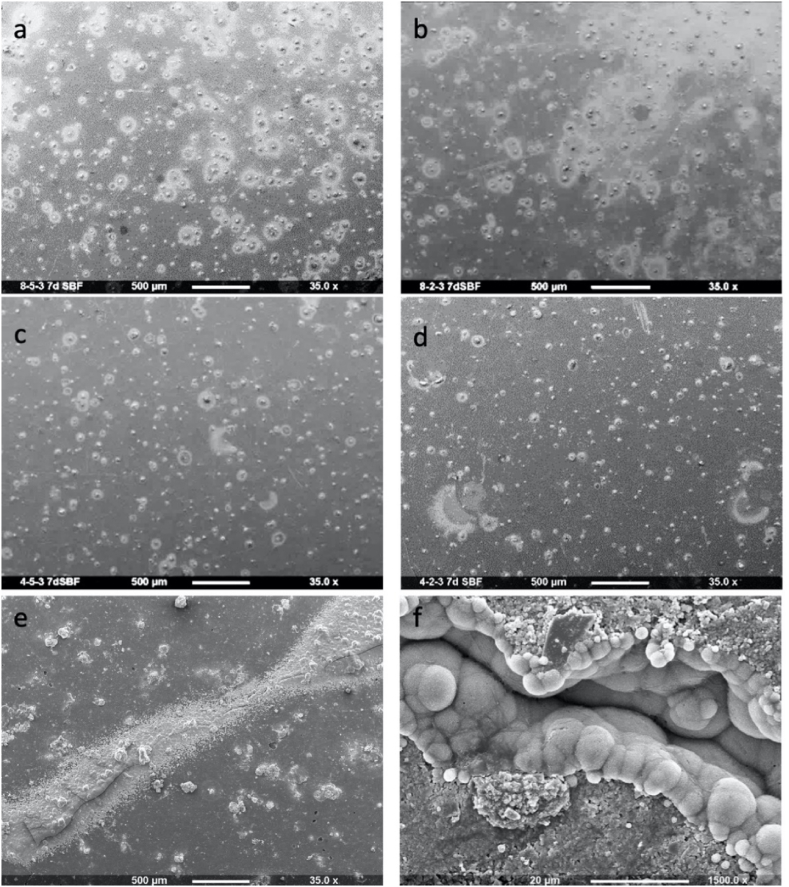
SEM images of a: F58, b: F28, c: F54, d: F24 discs after immersion in SBF for 7 days showing higher mineral deposits (light grey areas) with formulations of 8% MCP (a and b) in comparison to 4% MCP formulations (c and d). Minerals deposition was also detected at the fracture site of F54 discs that were partially fractured after 3 months in water. The mineral precipitation is observed following a further 2 months in SBF (e and f).

Upon increasing the level of MCP in the sample, an increase in mineral precipitation on the surfaces was clearly observed after 7 days (Fig. 5). Enhanced PLS level had no obvious effect. SEM images of fracture sites for the 3 month water stored F54 samples are provided in Fig. 5e and f. Following a further 2 months immersion in SBF, mineral could readily be observed within and around the fracture site but there was less on the remaining material surface.

## 4 Discussion

This study provides the mechanical data used to optimise levels of PLS and MCP in Renewal MI. These two components together promote water sorption from the tooth surfaces that improves the material unique property of being able to penetrate deeply into caries affected or demineralised dentine (Alkhouri et al., 2022; Alkhouri, 2020) . This material is currently undergoing a phase II efficacy trial at the Eastman Dental Institute. This was following a phase I safety trial that demonstrated suitability of Renewal MI to be placed on hand-excavated dentine. This demonstrated feasibility of using Renewal MI for minimally invasive restoration of children's teeth. Renewal MI has similar handling characteristics of a glass ionomer cement but is stronger and unlike the cements, able to penetrate and form very long resin tags in demineralised dentine (Alkhouri et al., 2022; Alkhouri, 2020) . The aim of this work was to determine what levels of PLS and MCP could be added whilst maintaining long-term mechanical properties above the ISO 4049 minimum of 80 MPa that is required for restorations in occlusal surfaces.

This study demonstrated that enhanced sample thickness significantly increased delay time before start of polymerisation of the experimental composites. Conversely, PLS and MCP levels had no significant effect. However, higher PLS or MCP significantly enhanced solubility. PLS caused a greater increase than MCP in water sorption and PLS release and more reduction in strength. Raised MCP enhanced mineral precipitation from SBF and enabled filling of cracks.

In comparison, Z250 had significantly lower delay time at high depth. Its lower final conversion, reduces polymerisation shrinkage but was close to a critical 50% level, below which monomer release is likely (Walters et al., 2016). Despite low conversion, Z250 solubility was barely detectable. Lack of hydrophilic components, high filler load and effective silane coupling would explain Z250 low water sorption and high strength.

Enhanced conversion of UDMA compared with BISGMA based composites (eg Z250) is well known (Walters et al., 2016). When combined with polypropylene glycol dimethacrylate (PPGDMA), UDMA polymerisation in the experimental materials can be further improved (Walters et al., 2016). The level of CQ (1 wt%) enables effective cure of this monomer system, without need for an additional, potentially cytotoxic, amine activator (Kangwankai et al., 2017).Matching of filler and resin refractive index reduces scattering thereby improving conversion at depth (Son et al., 2017).

With >50% conversion, unreacted cytotoxic monomers are less likely to leach out upon absorption of water (Goldberg, 2008). Furthermore, the crosslinking reaction can improve mechanical characteristics (Aljabo et al., 2015). A benefit of greater cure depth is that composites may be placed in fewer layers (bulk fill).

Low solubility is crucial in conventional composites as it is primarily due to release of unreacted monomers which can be allergenic and cytotoxic (Muller et al., 2017). Furthermore, high water sorption may bring down strength. Water sorption and solubility of Z250 were in good agreement with other studies (Misilli and Gonulol, 2017). Many of the experimental formulations, however, failed to achieve solubility values lower than those recommended by ISO 4049 standard (BSI, 2009). As conversions are high this is unlikely due to monomer release but caused by dissolution of PLS and MCP.

As with water sorption, PLS release from the modified Renewal MI formulations was initially proportional to the SQRT of time and therefore diffusion controlled. Higher PLS percentage release with greater composite PLS concentration could be due to enhanced water sorption and solubility enabling release from slightly deeper within the sample. Final release, of between 8 and 13%, suggests PLS is largely released from surface layers, on both top and bottom of the 1 mm thick discs. The trends with time and composite level are consistent with previous PLS release studies from similar formulations (Yaghmoor et al., 2020). The early levels of release seen in this study may be sufficient to kill low level planktonic *Strep. Mutans.* but have limited benefit when sucrose is present and biofilms form (Lygidakis et al., 2020; Yaghmoor et al., 2020). The sealing properties generated by PLS may, however, be more important in preventing recurrent disease.

Z250 flexural strength was comparable with those obtained previously (Thomaidis et al., 2013; Chung et al., 2004) and higher than for the experimental materials. Composite high strength is obtained by high filler content in addition to ensuring the matrix phase is well cured and effectively bound to the filler (Kangwankai et al., 2017). Poor wetting and interaction between the matrix and hydrophilic particles would explain low initial strength with Renewal MI formulations. With longer water immersion times, high water sorption caused by increased PLS could plasticise the matrix allowing greater deformation under load. It might also hydrolyse and damage bonds between matrix and the filler phases. Conversesly, MCP may crystallize as other species that bind water thereby having less detrimental effects on mechanical properties.

Apatite precipitation seen in this study was less than previously observed when MCP was combined with tricalcium phosphate (Kangwankai et al., 2017). The lack of later term precipitation would be consistent with formulations only releasing ions at early times or if the material is fractured. The influence of MCP content on remineralising is consistent with a previous study where higher MCP resulted in thicker layers of minerals covering the composite discs (Kangwankai et al., 2017). In a previous publication, Renewal MI has shown enhanced precipitation at the adhesion interface in comparison to commercial comparators (Glass ionomer cement and Activa) (Alkhouri et al., 2022). This property could potentially close any remaining gaps between the restoration and the tooth structure, remineralise any carious tissues and help fight against recurrent caries. Further tests are required to check the depth of minerals precipitation and whether this is significant enough to remineralised carious dentine.

With restoration of highly carious primary teeth in young children, antibacterial and remineralising composites would better support placement directly on affected dentine. In this study, higher values of MCP and PLS were 8 and 5 wt% as this previously gave close to maximum demineralised dentine sealing benefit (Alkhouri, 2020). Lower levels of 4 and 2 wt% enable easy detection of property variations with composition. Following these studies, MCP level in commercially manufactured Renewal MI was selected to be 8 wt% MCP to give higher remineralising potential. The PLS level was fixed at an intermediate value of 4 wt%. This was to ensure sufficient water sorption to provide good sealing whilst maintaining long-term strength above the ISO 4049 1 day requirement of 80 MPa.

## 5 Conclusions

Modified Renewal MI formulations with high or low PLS and MCP have high degree of monomer conversion following 40s of light-curing(up to 4 mm). Increasing PLS and MCP both raised solubility, whilst water sorption was primarily increased by the former. Higher PLS gives greater early PLS release whilst higher MCP promotes greater mineral precipitation. Whilst higher PLS caused a significant decline in biaxial flexural strength, doubling MCP did not.

## Funding

This work was supported by The 10.13039/501100000272National Institute for Health Research, 10.13039/501100000765University College London Hospitals, Biomedical Research Centre (NIHR UCL BRC: BRC522a/OH/AY/110380) (www.uclhospitals.brc.nihr.ac.uk); 10.13039/501100000272National Institute for Health Research (NIHR) under its Invention for Innovation (i4i) (NIHR: II-LB-0214-20002) (https://www.nihr.ac.uk); The 10.13039/501100000266Engineering and Physical Sciences Research Council (EPSRC: EP/I022341/1) (https://epsrc.ukri.org); 10.13039/100010269Wellcome Trust (ISSF/FHCE/0079) (https://wellcome.ac.uk). The views expressed are those of the author(s) and not necessarily those of the NHS, the NIHR or the Department of Health. The funders had no role in study design, data collection and analysis, decision to publish, or preparation of the manuscript.

## CRediT authorship contribution statement

**Nabih Alkhouri:** Writing – original draft, Methodology, Investigation, Formal analysis, Data curation. **Wendy Xia:** Writing – review & editing, Validation, Methodology, Funding acquisition, Conceptualization. **Paul Ashley:** Writing – review & editing, Validation, Supervision, Project administration. **Anne Young:** Writing – review & editing, Validation, Supervision, Project administration, Methodology, Funding acquisition, Formal analysis, Conceptualization.

## Declaration of competing interest

The corresponding author A.Y has two patents on the use of MCP and PLS in dental composites licensed to a dental company. N.A, W.X and P.A declare no competing interest.

## Data Availability

Data will be made available on request.
